# The role of redox mechanisms in hepatic chronic wound healing and fibrogenesis

**DOI:** 10.1186/1755-1536-5-S1-S4

**Published:** 2012-06-06

**Authors:** Erica Novo, Maurizio Parola

**Affiliations:** 1 Department of Experimental Medicine and Oncology, University of Torino, Corso Raffaello 30, 10125, Torino, Italy; 2 Interuniversity Centre for Liver Pathophysiology, University of Torino, Corso Raffaello 30, 10125, Torino, Italy

## Abstract

Under physiological conditions, intracellular and tissue levels of reactive oxygen species (ROS) are carefully controlled and employed as fine modulators of signal transduction, gene expression and cell functional responses (redox signaling). A significant derangement in redox homeostasis, resulting in sustained levels of oxidative stress and related mediators, plays a role in the pathogenesis of human diseases characterized by chronic inflammation, chronic activation of wound healing and tissue fibrogenesis, including chronic liver diseases. In this chapter major concepts and mechanisms in redox signaling will be briefly recalled to introduce a number of selected examples of redox-related mechanisms that can actively contribute to critical events in the natural history of a chronic liver diseases, including induction of cell death, perpetuation of chronic inflammatory responses and fibrogenesis. A major focus will be on redox-dependent mechanisms involved in the modulation of phenotypic responses of activated, myofibroblast-like, hepatic stellate cells (HSC/MFs), still considered as the most relevant pro-fibrogenic cells operating in chronic liver diseases.

## Introduction

### Basic concepts on oxidative stress and related reactive mediators

**Figure 1:**
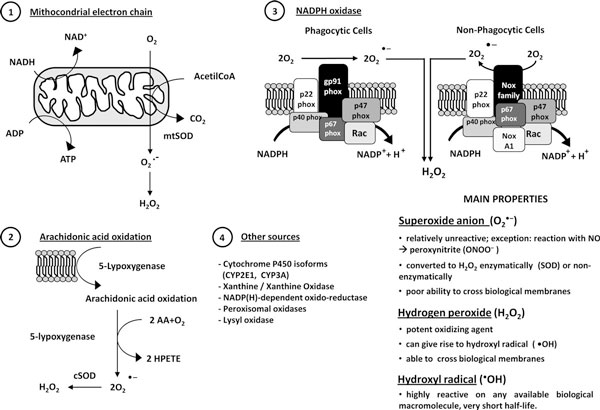
**Intracellular sources and main properties of ROS**. Within the cell ROS can be generated from different sources as follows: 1) as O_2_^•- ^released at the level of complex I and III of mitochondrial electron chain reaction, with O_2_^•- ^being immediately dismutated to H_2_O_2 _by mtSOD; 2) as O_2_^•- ^following the reaction of 5-lypoxygenase on arachidonic acid, then dismutated to H_2_O_2 _by cSOD; 3) as O_2_^•-^, then dismutated into H_2_O_2_, following activation (i.e., ligand-receptor interaction) of NADPH oxidase in either phagocytic or non-phagocytic cells; 4) as different ROS generated following the redox reactions catalyzed by a number of cytochromes and enzymes. Critical properties of major ROS are also briefly summarized.

**Figure 2:**
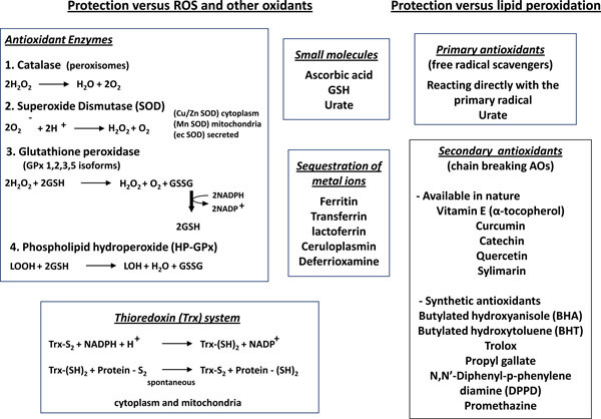
**Major antioxidant defences in mammalian cells**. A brief overview of antioxidant defences is offered. Antioxidant defences have been subdivided in those having as a major goal to display protection versus ROS and other oxidants (left panels) and in those (including both naturally occurring and synthetic molecules) able to prevent the chain reactions of lipid peroxidation (right panels).

Aerobic organisms use molecular oxygen (O_2_) as the final electron acceptor for mitochondrial cytochrome c oxidase that, as terminal functional element of mitochondrial NADH dehydrogenase enzyme complex, catalyzes the four electron reduction of O_2 _[1]. During mitochondrial oxidative phosphorylation and other electron transfer reactions partially reduced and highly reactive oxygenspecies (ROS) are generated, including superoxide anion (O_2_^•-^), hydrogen peroxide (H_2_O_2_) and hydroxyl radical (^•^OH), that at very high levels can result in cell injury and death, according to their properties and intracellular sources (resumed in Figure 1). In order to survive, aerobic organisms have developed evolutionary conserved mechanisms and strategies (including antioxidant defences resumed in Figure 2, see ref. [5] for more details) to carefully control generation of ROS and other oxidative stress - related mediators to maintain intracellular redox homeostasis and to use these reactive intermediates to modulate signal transduction, gene expression and cellular responses (i.e., redox signaling) [1-5]. At present, it is widely accepted that increased and/or sustained levels of ROS and other mediators of oxidative stress play a significant role in atherosclerosis, diabetes, cardiovascular diseases, cancer, neuro-degenerative diseases as well as in chronic inflammatory and fibrogenic diseases involving chronic activation of wound healing, including chronic liver and lung diseases.

### Generation of relevant oxidative stress mediators

In addition to major ROS like O_2_^•-^, H_2_O_2 _and ^•^OH one should remember that other reactive mediators of oxidative stress are represented by end-products of lipid peroxidation (LPO), a complex chain reaction initiated by a ROS or other free radicals with polyunsaturated fatty acids of membrane phospholipids, leading to their oxidative degradation, and exacerbated by the presence of divalent metal ions [5,6]. End-products of LPO are represented by reactive aldehydes including malonyldialdehyde (MDA) and 4-hydroxy-2,3-alkenals (HAKs) of different chain length as well as by F_2_-isoprostanes that derive mainly by nonenzymatic peroxidation of arachidonic acid [5,6]. Both 4-hydroxy-2,3-nonenal (HNE), the most active HAK in biology and pathophysiology [5], as well as F_2_-isoprostanes are relatively stable and lipophilic compounds that can diffuse from the site of generation and easily cross biological membranes to exert both cytotoxic and signalling action. Their detection in biological fluids or tissues is considered as a reliable way to evaluate "in vivo" oxidative stress [5].

Nitric oxide (NO), a small hydrophobic molecule that crosses cell membranes without needing channels or receptors, is generated in living organisms by several isoforms of NO synthases (NOS), including one mitochondrial, that are able to produce NO through the conversion of L-arginine in citrullin. NO has a role in controlling vascular tone, cellular adhesion, vascular permeability and inhibition of platelet adhesion but pathologic effects comes from oxidation products, included in the definition of reactive nitrogen species (RNS) such as the powerful oxidant peroxynitrite (ONOO^-^) which is formed by the rapid reaction between NO and O_2_^•-^. When produced in excess, peroxynitrite can oxidize any cellular constituent, leading to disruption of cell signalling pathways and to the induction of either necrotic or apoptotic cell death [7].

### Reactions of ROS, HNE and peroxynitrite

ROS can interact with any biological macromolecule [5,8]: a) DNA, leading to oxidative damage, strand breaks or adduct formation (like 8-hydroxy-deoxy-Guanidine or 8-OH-dG); b) lipids, by eliciting lipid peroxidation and subsequent degradation and fragmentation; c) proteins, leading to oxidation of critical residues (-SH groups), formation of intra-molecular disulfide bonds, thiol/disulfide changes, formation of di-tyrosine and of protein cross linking, or even ubiquitination and proteasomal degradation. Similar reactions have been described for ONOO^- ^that can, in addition, also lead to nitrosation or nitration of proteins. HNE can exert its cytotoxic and signalling action by forming Michael type adducts on lysine, cysteine or histidine residues as well as, by reacting with DNA, lead to adduct formation, strand breaks and genotoxicity [5,6].

### Redox homeostasis and redox signalling

Redox signalling is a general definition that can be used to indicate any condition, in physiology or pathophysiology, in which a process can be regulated or modulated by a signal that is delivered through redox chemistry [2-5,9]. The concept of redox homeostasis is intrinsically simple if one translates this definition into a theoretical condition of cells or tissues that are not exposed to ROS. When, by any means or sources, significant levels of ROS are generated in a biological system (i.e., altering redox homeostasis) "redox signalling" is then representing the response or part of the response designed to "reset" the original state of equilibrium.

### Redox homeostasis

Redox homeostasis is primarily granted or controlled by highly specialized enzymes like catalase, thioredoxins, SODs and GPXs as well as by naturally occurring antioxidants like GSH, vitamin E, β-carotene, ascorbate, urate, and many others. The antioxidant defences (see Figure 2) are further implemented by less specific, but much more abundant, reactants like aminoacids, peptides and proteins. In practical terms, cells in which very low levels of ROS are generated do not suffer a significant unbalance of pro-oxidants vs antioxidant defenses and then do not respond by means of a redox signalling [2-5,9,10]. Depending on the rise in intracellular levels of ROS, the following alternatives can apply: a) low and/or transient increase in ROS or other mediators will lead to a time-limited shift in redox balance and redox signalling will operate through defined redox-sensitive signalling pathways and transcription factors to up-regulate genes carrying ARE (antioxidant responsive element) sequences coding for antioxidant enzymes in order to reset in the due time redox homeostasis; b) too high levels of ROS and oxidants (severe oxidative stress) can lead to irreversible damage to cell structures and functions causing cell death; c) increased and persistent levels of oxidative stress, not overtly able to induce cell death, will result in a shift of the intracellular redox state to a different, chronically deregulated state, in which redox signalling is up-regulating different patterns of target genes and cell responses that can sustain the development of chronic diseases [2-5]. A note to this didactic scenario: in a tissue undergoing chronic injury, inflammation and wound healing the three conditions are likely to coexist and then one can envisage an overall scenario in which the pathological condition is resulting from the sum of both ROS-dependent damaging effects and changes in gene expression.

### Redox sensors and redox signalling

**Figure 3:**
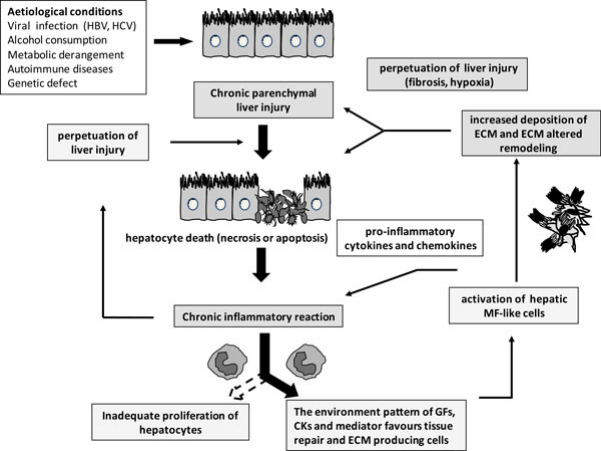
**Major events involved in the fibrogenic progression of chronic liver diseases**. CLDs may involve different aetiological agents or conditions able to cause persisting (i.e., chronic) parenchymal liver injury and then hepatocyte cell death (either necrosis or apoptosis). As a result of chronic liver injury, a persistent inflammatory reaction can occur which may significantly affect the progression of the disease by either contributing to the perpetuation of parenchymal injury or by generating a microenvironment pressure in terms of growth factor, cytokine and other mediators that can efficiently favour tissue repair and the activation of pro-fibrogenic cells. This is the scenario that will lead to the persistent activation of myofibroblast-like cells from different precursors. Activated MFs will contribute either to perpetuation of inflammation by releasing pro-inflammatory mediators or to the excess synthesis and accumulation of fibrillar (rich in collagen type I and III) extracellular matrix (ECM) components. If the aetiological agent or causal condition persists, the CLD can undergo a fibrosclerotic progression to cirrhosis and liver failure. Since cirrhosis is characterized by formation of regenerative nodules of parenchyma surrounded and separated by fibrotic septa, this scenario is intrinsically associated with significant changes in hepatic angio-architecture. Fibrosis itself as well as the development of hypoxic areas have been reported to further contribute to perpetuation of liver injury.

A redox sensor is a redox-sensitive specialized protein, that is able to "sense" intracellular levels of ROS by a redox-based mechanism affecting one or more residues/domains within its three-dimensional structure, then transforming the redox change into a specific "signal" able to positively affect signalling pathways (particularly those elicited by interaction of growth factors, cytokines, chemokines and other peptides with their respective receptors) and transcription of redox-sensitive genes. In conditions of chronic liver diseases (see major events in Figure 3) the following mechanisms and concepts are likely to have a major role (for more details see ref. [2-5,9,10]).

a) Reinforcement of signal transduction elicited by peptide ligands (PDGF, VEGF, Angiotensin II, MCP-1, TGFβ1, TNF) produced in the scenario of chronic liver diseases as a consequence of interaction with their corresponding membrane receptors. ROS production in non-phagocytic cells (as hepatic myofibrobasts) can be elicited through receptor tyrosine kinase- and Rac-mediated activation of NADPH-oxidase or by involving different types of receptors and signalling components (TGFβ1 operates through receptor serine/threonine kinase and involves Smads and Src kinases).

b) ROS, generated within the cells or entering from outside, can enhance signalling pathways by inhibiting protein tyrosine phosphatases (PTPs) usually through reversible oxidation of critical residues (mainly -SH groups); prevention of de-phosphorylation can then reinforce down-stream signal transduction of those pathways [5,9-11].

c) Intra- and extracellular ROS can activate protein kinases as well as MAPK cascades in the cytoplasmic compartment. This has been described, through mild oxidation or mild shift in the intracellular thiol/disulfide redox state, for components belonging to the Src family of protein tyrosine kinases (for example p59^fyn ^and p56^lck^) or JAK2, c-Jun NH_2_-terminal kinases (JNKs), p38^MAPK^, ERK-1 and ERK-2 (the latter not in all cell types) or some PKC isoform [2-5,9,10]. Concerning JNKs, two activating mechanisms have been proposed relying on redox-activation of the upstream kinase ASK1 or the inhibition of specific JNK-phosphatases [12,13].

d) ROS and oxidative stress can activate defined transcription factors and the best characterized examples are NF-kB and AP-1. NF-kB, in particular, is known to be involved in inflammatory reactions, in the control of cell growth and in prevention of apoptosis (possibly also of necrotic cell death) as well as in maintaining mitochondrial integrity and as a regulator of antioxidant activity [14-16]. In a chronic inflammatory environment, such as the one of CLDs, a general message is that all cytokines leading to NF-kB activation are likely to cause intracellular generation of ROS that are then responsible for IKK activation and IκBα degradation. Once again, ROS produced within cells as a part of the response induced by inflammatory cytokines contribute to reinforce the signal and modulate the overall response and fate of the target cells.

### Oxidative stress and redox signaling in chronic liver diseases

As indicated in Figure 3, there are several events that are relevant for fibrosclerotic progression of CLDs and that could be considered relatively independent on the specific aetiology, including persisting hepatocyte necrotic or apoptotic cell death, persisting inflammatory response and chronic activation of wound healing response. Comprehensive reviews on the pathogenesis of CLDS and, particularly, liver fibrogenesis and the role of hepatic myofibroblasts (MFs) are available for the interested reader which emphasize the role of oxidative stress (unequivocally detected in all clinical conditions as well as animal models of fibrogenic CLD) and related mediators [16-18], as well as the redox-related exacerbating (i.e., pro-fibrogenic) role of specific factors related or not to the aetiology (viral infection, alcohol consumption, cholestasis, metabolic alterations in NAFLD/NASH, iron overload, hypoxia) [5,19]. Here just a selected number of relevant redox-dependent mechanisms and events will be briefly resumed.

### Oxidative stress and parenchymal injury

Whether natural history and progression of CLDs are concerned, whatever the aetiology, persisting liver injury and hepatocyte loss predominate and, indeed, severe oxidative stress can be considered as a major cause for both necrotic and apoptotic cell death of parenchymal cells [5,20-22] either resulting from inflammatory flares (i.e., HCV or HBV infection) through increased ROS generation by leukocytes and/or following ethanol consumption, hepatic iron overload, down-regulation of antioxidant status, to name just a few conditions. The following major messages should be recalled [5,20-26]: a) severe oxidative stress may lead to both hepatocyte necrosis and apoptosis, with necrosis mainly resulting from irreversible mitochondrial damage and/or inactivation of executioner caspases; b) both necrosis and apoptosis can be found on the same section, in association with the other events of the chronic scenario (inflammation, fibrogenesis, angiogenesis, etc.); c) increased levels of ROS may be critical in deciding whether the target cell may survive or die, as described for the engagement of death receptors (DR) or Toll-like receptors (TLR) by respective ligands and the involvement of the critical kinase RIP (from Receptor Interacting Protein) [23-26]; d) ROS-related sustained activation of JNK isoforms is a well characterized event leading to cell death in several conditions; moreover, in hepatocytes NF-kB inhibition sensitize cells to TNF-induced apoptosis by means of JNK sustained activation [27]; e) ROS-related mitochondrial damage is a typical example of two way-injury since mitochondria can represent not only a source of ROS (particularly when their integrity is deranged) but also a target for their action in relation to cell death [26]; f) ROS are critical in mediating cell death of fatty hepatocytes due to excess of free fatty acids (FFAs) in the liver of NAFLD and NASH patients; this may happen in FFAs-related up-regulation of TNF, increased Fas ligand binding to Fas (CD-95) or induction of endoplasmic reticulum (ER)-stress and the so called "unfolded protein response" (UPR) [5,28]; g) ER-stress, and then ROS, have been implicated also in hepatocyte apoptosis in chronic hepatitis C and ALD [5]; h) NO and related RNS can theoretically promote or prevent apoptotic cell death by interfering with either mitochondrial-dependent or -independent signalling pathways [5].

### Hepatic myofibroblasts survive to oxidative stress

Human hepatic MFs can survive to ROS, HNE and other pro-oxidants [29] and this relies on the MFs activation-related specific "survival attitude" involving up-regulation of Bcl2, over-activation of pro-survival pathways, including NF-kB-related ones, and down-regulation of Bax [30,31]. Hepatic MFs can then survive to conditions of oxidative stress usually operating in CLDs that, rather (see later), are more likely to sustain their pro-inflammatory and pro-fibrogenic responses.

### Oxidative stress and inflammatory response

Mediators of oxidative stress, whatever the source, aetiology or metabolic condition, are involved in the up-regulation or modulation of the expression of pro-inflammatory cytokines and chemokines by different cells, (including inflammatory cells as well as HSC/MFs or, presumably, MF-like cells, mostly through activation of NF-kB [2-5]. In addition, the following major concepts may be recalled [5]: a) ROS are involved in the process of phagocytosis, possibly by leading to amplification of the stimulating signal that follows engagement of Fc receptors on the surface of phagocytic cells; b) ROS may have a role in apoptosis - related removal of leukocytes during inflammatory responses; c) HNE as well as other 4-hydroxy-2,3-alkenals (HAKs), have been reported to be able to stimulate leukocyte chemotaxis at very low concentrations; d) ROS and HNE elicit in vivo and in vitro up-regulation of the chemokine MCP-1, then sustaining recruitment/activation of monocytes/macrophages and Kupffer cells as well as attracting also HSC/MFs [5,32,33].

### Oxidative stress and related mediators sustain pro-fibrogenic action of MFs

**Figure 4:**
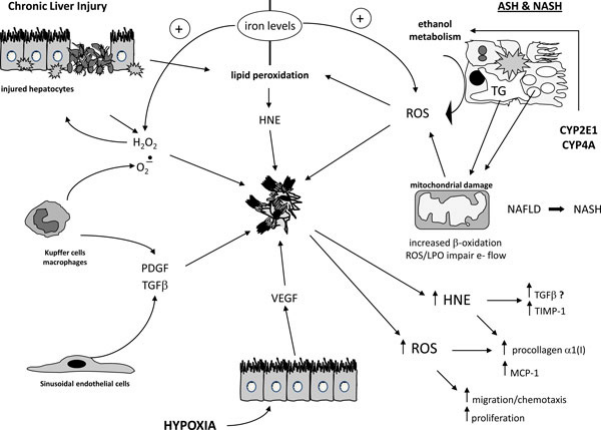
**ROS and related mediators as pro-fibrogenic stimuli for hepatic myofibroblasts**. Activated hepatic stellate cells (HSC/MFs) and/or other populations of hepatic MFs may be envisaged as putative "target" cells that modify their behavior and phenotypic responses when exposed to the action of ROS and other oxidative stress - related reactive intermediates (for example, 4-hydroxynonenal or HNE, a major aldehydic end-product of lipid peroxidation elicited through different mechanisms). ROS and the other reactive intermediates can be formed following damage to hepatocytes, activation of either resident kupffer cells or macrophages recruited from peripheral circulation, release from damaged mitochondria, generation following activation of certain cytochrome P450 isoforms (as in chronic ethanol-mediated injury or in condition of NAFLD and NASH), to name just the most relevant options. More details can be found in the text.

Literature data of the last two decades have outlined that activated, MF-like, hepatic stellate cells (HSC/MFs) and, likely, MFs of different origin, are ideal pro-fibrogenic targets for ROS and HNE (Figure 4)[5,16-18]. Best characterized mechanisms and concepts are the following: a) antioxidant supplementation can prevent or reduce liver fibrosis in experimental models; b) ROS and HNE exert a direct, paracrine pro-fibrogenic action on human HSC/MFs by up-regulating pro-collagen type I expression, although through different signalling pathways [5], and the same event follows intracellular generation of ROS by TGFβ1 and leptin [34]; c) intracellular generation of ROS occurs in HSC/MFs and hepatic MFs in association to cytokine-receptor interactions and parallel activation of NADPH-oxidase [5,10,35], revealing a novel putative direct or indirect (as the case of losartan for AT-1 receptor) target for therapy in CLDs; d) increased intracellular levels of ROS, whatever the cause (cytokine-receptor interaction, entry from extracellular environment, increased mitochondrial release under hypoxic conditions, following iron overload or ER stress) is sufficient to stimulate oriented migration in target pro-fibrogenic cells through a biphasic mechanism [36-38]; e) intracellular generation of ROS is emerging as a common mechanism able to mediate the pro-angiogenic action of PDGF-BB and leptin on human HSC/MFs [39,40]; f) the specific mediator makes the difference, with ROS being able to up-modulate MFs proliferation [5] and chemotaxis [29] and HNE having no effect on migration or even able to inhibit PDGF-dependent proliferation by specifically targeting PDGF-βR tyrosine kinase [41].

## Conclusions

The final response of a target cell to oxidative stress and related mediators may be relatively unpredictable and significantly affected by the steady state concentration of reactive species, the intrinsic state of the target cell (i.e., activated versus quiescent), the presence of specific growth factors and cytokines in the microenvironment as well as of other cells able to generate ROS or HNE as well as the concomitant generation of NO in the microenvironment. Although severe oxidative stress may contribute to the progression of CLDs by eliciting parenchymal cell death, a significant derangement in redox homeostasis, resulting in sustained levels of oxidative stress and related mediators, is believed to play a major role in sustaining chronic inflammatory response as well as chronic activation of wound healing, with hepatic MFs representing ideal target cells exhibiting not only pro-fibrogenic but also pro-inflammatory and pro-angiogenic properties.

## List of abbreviations used

ARE: antioxidant responsive element; CLDs: chronic liver diseases; DR: death receptors; ER: endoplasmic reticulum; ERK: extracellular signal-regulated kinases; FFAs: free fatty acids; HAKs: 4-hydroxy-2,3-alkenals; HBV: hepatitis B-virus; HCV: hepatitis C-virus; HNE: hydroxy-2,3-nonenal; H_2_O_2_: hydrogen peroxide; JNKs: c-Jun NH_2_-terminal kinases; LPO: lipid peroxidation; MCP-1: monocyte chemoattractant protein-1; MDA: malonyldialdehyde; MFs: myofibroblasts; NAFLD: non-alcoholic fatty liver disease; NASH: non-alcoholic steatohepatitis; NF-kB: nuclear factor-kappa B; NO: nitric oxide; NOS: NO synthases; O_2_^•-^: superoxide anion; ^•^OH: hydroxyl radical; 8-OH-dG: 8-hydroxy-deoxy-Guanidine; ONOO^**-**^: peroxynitrite; RNS: reactive nitrogen species; PDGF: plateled derived growth factor; PTPs: protein tyrosine phosphatises; RIP: Receptor interacting protein; ROS: reactive oxygen species; TGFβ1: transforming growth factor β1; TLR: Toll-like receptors; TNF: tumor necrosis factor; UPR:unfolded protein response; VEGF: vascular growth factor.

## Competing interests

The authors declare that they have no competing interests.
